# Attention-Deficit/Hyperactivity Disorder and Suicidal Risk in Major Depression: Analysis of 141,530 Adolescent Hospitalizations

**DOI:** 10.7759/cureus.7949

**Published:** 2020-05-04

**Authors:** Shaheer Zahid, Krishna Priya Bodicherla, Noha Eskander, Rikinkumar S Patel

**Affiliations:** 1 Psychiatry, Saint James School of Medicine, Park Ridge, USA; 2 Psychiatry, Sri Devaraj Urs Medical College, Kolar, IND; 3 Psychiatry, Ain Shams University Hospital, Cairo, EGY; 4 Psychiatry, Griffin Memorial Hospital, Norman, USA

**Keywords:** child and adolescent psychiatry, depression, mdd, major depressive disorder, suicide prevention, suicide risk, attention deficit hyperactivity disorder (adhd)

## Abstract

Objectives

We conducted a cross-sectional study to understand the differences in demographics and psychiatric comorbidities in adolescents with major depressive disorder (MDD) and assess the risk of suicidality due to comorbid attention-deficit/hyperactivity disorder (ADHD).

Methods

We included 141,530 adolescents (age, 12 to 18 years) with a primary diagnosis of MDD from the nationwide inpatient sample (NIS, 2012-2014), and grouped by a comorbid diagnosis of ADHD (N = 22,665, 16%). Logistic regression analysis was used to measure the demographic predictors for ADHD in adolescents with MDD, and to measure the suicidal risk in ADHD versus non-ADHD.

Results

Comorbid ADHD was prevalent in whites (71.9%), and males had two times higher odds (95% CI 2.25-2.41) compared to females. The most prevalent comorbidities seen in ADHD-cohort were anxiety disorders (46.3%) and substance abuse (20.1%) with 1.3 times higher odds of substance abuse (95% CI 1.41-1.65) compared to non-ADHD. Suicidal behaviors were seen in a higher proportion of the ADHD cohort compared to the non-ADHD cohort (54.3% vs. 52.7%). ADHD and suicidal behaviors relationship was statistically significant but had a very small positive association (OR 1.04, 95% CI 1.01-1.08) after controlling for demographic confounders and comorbidities. There was a significant increase in the number of MDD hospitalization with ADHD for suicidal behaviors from 51.1% (N = 3,360) in 2012 to 58.2% (N = 5,115) in 2014.

Conclusion

There exists a significant but small positive association between suicidal behaviors and comorbid ADHD in MDD adolescents. The suicide rate has increased by 52.2% during the study period in depressed adolescents with ADHD. This calls for early diagnosis and management of ADHD and early-onset depression in adolescents to prevent suicide risk.

## Introduction

Depression among adolescents is common psychopathology which may often go unrecognized [1]. With almost one in every 10 adolescents experiencing a major depressive disorder (MDD), an estimated 2.3 million adolescents in 2017 experienced a depressive episode that caused severe functional impairment in daily lives [2]. The prevalence is higher among females compared to males with a sharp increase seen after puberty and at the end of adolescence [3].

One of the major concerning factors seen in depressed individuals is the risk for suicide as it is the foremost cause of increased mortality rates in the United States (US). According to the center of disease control and prevention (CDC), suicide is among the second leading cause of mortality in adolescents [4]. And throughout the years, there has been a steady increase in suicide rate in the general population, with the highest risk seen in men. Suicide has a major impact on the US economy with 50.8 billion US dollars spent on healthcare costs in 2013 [4].

Attention-deficit/hyperactivity disorder (ADHD) is one of the most prevalent pediatric psychiatric illness. As per a national parent survey published in 2016, about six million (9.4%) children have been diagnosed with ADHD, with the majority of the children ranging between the ages of six to 17 years [1]. ADHD may have an impact on suicidal ideations among adolescents but there exists no direct correlation between increased suicide risk in depressed adolescents [5]. A study conducted by Lan et al. in Taiwan found that ADHD increases the risk of suicidal ideation in adolescents [6]. There exists a link between higher impulsiveness in ADHD patients increasing the suicide risk among college students, and also the inability to appropriately regulate emotions leading to outbursts [7, 8]. Over the years, with an improved diagnostic formulation for ADHD and a better understanding of early-onset depression in adolescents, a better treatment plan can be developed to cater the psychiatric care [9].

In our study, we used the national inpatient data to understand the differences in demographics and psychiatric comorbidities in adolescents with MDD by comorbid ADHD, and assess the risk of suicidal behaviors with comorbid ADHD versus non-ADHD. Also, we studied the trend in adolescent inpatients with MDD and comorbid ADHD in the US hospitals.

## Materials and methods

Data source

We conducted a retrospective data analysis using the nationwide inpatient sample (NIS, 2012 to 2014) from the healthcare cost and utilization project (HCUP). The NIS provides inpatient data records from about 4,400 non-federal hospitals across 44 states in the US [10]. Diagnostic information in the NIS is identified using the International Classification of Diseases, ninth edition (ICD-9) codes and clinical classification software (CCS) codes [11].

Inclusion criteria and outcome variables

We included patients (age 12 to 18 years) with a primary discharge diagnosis of MDD using the ICD-9 codes 296.20-296.26 or 296.30-296.36 and further grouped by secondary discharge diagnosis of ADHD using the ICD-9 codes 314.00 or 314.01.

Demographic variables studied included age, sex (male or female), and race (White, Black, Hispanic, and Asian/native American) [11]. The comorbid diagnosis for impulse control disorders, anxiety disorders, psychotic disorders, suicidal behaviors, alcohol abuse, and substance abuse was identified using ICD-9 diagnosis codes and CCS codes [11].

Statistical analysis

We used cross-tabulation and descriptive statistics to discern the demographic and comorbidities differences in MDD by comorbid ADHD diagnoses. Logistic regression analysis was used to measure the demographic predictors for ADHD in adolescents with MDD, and to measure the risk of suicidal behaviors in ADHD versus non-ADHD cohorts after controlling for demographic confounders and psychiatric comorbidities. We used the linear-by-linear association test for measuring the differences in demographics and comorbidities, and the ANOVA test for measuring the changes seen in mean age over the study period in adolescents with MDD and comorbid ADHD. A P-value of less than 0.05 was used to determine the statistical significance and all analysis was done using the statistical package for the social sciences (SPSS) version 26 (IBM Corporation, Armonk, NY).

Ethical approval

Individual identifiers were used to protect the patient's identity and health-related information. So, the use of de-identified NIS database in this study does not require approval from the institutional review board [10].

## Results

We had a sample of 141,530 adolescent inpatients with a primary diagnosis of MDD, and 16% (N = 22,665) had comorbid ADHD. A higher proportion of the ADHD cohort were females (56.8%), but when compared with the non-ADHD cohort, males had 2.3 times higher odds (95% CI 2.25-2.41, P < 0.001) for comorbid ADHD compared to females. While comparing different races/ethnicities, comorbid ADHD was prevalent in Whites (71.9%), and there exists statistically non-significant difference when compared with Blacks (P = 0.271), while Hispanics and native Americans/Asians were having lower odds. The prevalence of comorbid ADHD increased with median household income, but there was no statistically significant difference in the relationship between household income and the co-diagnosis of ADHD.

The most prevalent comorbidities seen in the ADHD cohort were anxiety disorders (46.3%) and substance abuse (20.1%). Patients in the ADHD cohort had 1.5 times higher odds (95% CI 1.41-1.65, P < 0.001) of comorbid impulse control disorder, and 1.3 times for comorbid substance abuse (95% CI 1.41-1.65, P < 0.001. Suicidal behaviors were seen in a higher proportion of the ADHD cohort compared to non-ADHD (54.3% vs. 52.7%). ADHD and suicidal behaviors relationship was statistically significant but had a very small positive association (OR 1.04, 95% CI 1.01-1.08, P = 0.011) after controlling for demographic confounders and comorbidities as shown in Table 1.

**Table 1:** Demographic predictor and risk of comorbidities in depressed adolescents with ADHD ADHD: Attention-deficit/hyperactivity disorder

Variable	Major depressive disorder		Logistic regression analysis		
	ADHD (-)	ADHD (+)	Odd ratio	95% Confidence interval	P-value
Total N	118865	22665	-	-	-
Mean age, years	15.3	15.1	0.88	0.87 – 0.89	<0.001
Sex, %					
Female	74.2	56.8	Reference		
Male	25.8	43.2	2.33	2.25 – 2.41	<0.001
Race, %					
White	64.8	71.9	Reference		
Black	11.3	11.7	0.97	0.92 – 1.02	0.271
Hispanic	15.2	9.8	0.56	0.53 – 0.59	<0.001
Native American/Asian	8.7	6.5	0.69	0.65 – 0.74	<0.001
Median household income in percentiles, %					
0 – 25^th^	23.8	23.5	1.00	0.96 – 1.05	0.997
26^th^ – 50^th^	25.2	25.2	0.96	0.91 – 1.00	0.053
51^st^ – 75^th^	25.5	25.1	0.96	0.92 – 1.00	0.060
76^th^ – 100^th^	25.4	26.3	Reference		
Comorbidities, %					
No comorbidity	-	-	Reference		
Impulse control disorders	2.9	4.6	1.52	1.41 – 1.65	<0.001
Anxiety disorders	44.5	46.3	1.12	1.08 – 1.16	<0.001
Psychotic disorders	1.4	1.4	1.04	0.92 – 1.19	0.515
Suicidal behaviors	52.7	54.3	1.04	1.01 – 1.08	0.011
Alcohol abuse	6.1	6.9	1.04	0.96 – 1.11	0.348
Substance abuse	16.8	20.1	1.27	1.22 – 1.33	<0.001

The prevalence of comorbid ADHD increased in females from 50.8% in 2012 to 60.4% in 2014, while there is a decrease of 9.6% in the male population (P < 0.001). There is statistically no significant difference seen across the race during the study period. In terms of median household income, there is a bimodal change as there is an increase in prevalence in low-income families below the 25th percentile (20.1% to 23.6%) and a decrease in high-income families above 75th percentile (29.3% to 25.8%) during the study period. There was a significant increase of 52.2% in number of MDD hospitalizations with ADHD for suicidal behaviors (N = 3,360 and 51.1% in 2012 to N = 5,115 and 58.2% in 2014, P < 0.001). Among comorbidities, the highest increase in prevalence was seen in anxiety disorder (by 8.3%) and impulse control disorders (by 0.7%). There was a statistically significant decrease in alcohol abuse and substance abuse as shown in Table 2.

**Table 2:** National trend in adolescent inpatients with major depression and comorbid ADHD, 2012 to 2014 ADHD: Attention-deficit/hyperactivity disorder

	2012	2013	2014	Total	P-value
Total N					
Mean age, years	15.22	15.03	15.07	15.10	<0.001
Sex, %					
Female	50.8	58.0	60.4	56.8	<0.001
Male	49.2	42.0	39.6	43.2	
Race, %					
White	72.5	71.5	71.9	71.9	0.712
Black	11.6	12.0	11.5	11.7	
Hispanic	9.5	9.0	10.7	9.8	
Native American/Asian	6.4	7.5	5.9	6.5	
Median household income in percentiles, %					
0 – 25^th^	20.1	26.4	23.6	23.5	<0.001
26^th^ – 50^th^	25.8	23.6	26.0	25.2	
51^st^ – 75^th^	24.8	25.9	24.6	25.1	
76^th^ – 100^th^	29.3	24.1	25.8	26.3	
Comorbidities, %					
Impulse control disorders	4.3	4.5	5.0	4.6	0.040
Anxiety disorders	42.7	44.0	51.0	46.3	<0.001
Psychotic disorders	1.1	1.6	1.6	1.4	0.009
Suicidal behaviors	51.1	52.5	58.2	54.3	<0.001
Alcohol abuse	8.1	7.5	5.4	6.9	<0.001
Substance abuse	22.1	20.5	18.3	20.1	<0.001

## Discussion

Using a national sample from thousands of hospitals in the US, we were able to find a statistically significant but smaller positive association with suicidal behaviors due to ADHD in adolescents with MDD after controlling for demographic confounders and psychiatric comorbidities including substance abuse.

Based on a study conducted by Danielson et al., ADHD was prevalent among adolescents with three million being diagnosed with ADHD in 2016 [9]. So, we conducted the study in this age group with a mean age of 15.3 years. Boys were diagnosed with ADHD more often and we found that depressed adolescent males have two times higher likelihood for co-diagnosis of ADHD compared to females [9]. This possibly could be due to more behavioral symptoms and hyperactive and/or impulsive symptoms seen more in boys due to which parents seek psychiatric treatment more often [12]. Comorbid ADHD was more common in White adolescents from high-income families. Morgan et al. also found that compared to Whites, minority children including Blacks and Hispanics had lower odds for ADHD diagnosis [13].

We found that depressed adolescents with ADHD have a higher risk of comorbid impulse control disorder and anxiety disorders. Both these disorders are caused by a neurochemical imbalance in serotonergic and dopaminergic systems that slow down inhibitory responses leading to impulsive behavior, and difficult to inhibit intrusive thoughts causing increased anxiety [14, 15]. Substance abuse can lead to under-development of the brain leading to improper impulse regulation and communication failure among peers [16]. Also, a study by Patel et al. found that cannabis use in ADHD adolescents was associated with longer hospitalization stay and cost with decreased utilization of psychotropic regimen [17]. In our study, adolescents with MDD and ADHD had two times higher risk of substance abuse compared to those without ADHD.

There was a significant increase in suicidal behaviors among depressed adolescents with ADHD by 52.2% during the study period. Daviss found that MDD and ADHD were independent factors that increase suicide risk among adolescents though there was no correlation between both disorders [18]. Past studies found that ADHD was a significant predictor for suicidal ideation in adolescents especially in younger teens with substance abuse [16, 19]. So, in our study, we controlled the regression model with demographic confounders and various psychiatric comorbidities and found that comorbid ADHD is associated with a small positive association with suicidal behaviors in depressed adolescents.

Our study had some limitations. Firstly, the NIS lacks patient-level information that is required for evaluating the causal association between suicidal behavior and ADHD in MDD adolescents. Additionally, such cross-sectional database studies are subject to selection bias and moderate sensitivity of diagnostic codes for psychiatric conditions. Yet, using the NIS we were able to draw a population-based perspective on suicidal association due to ADHD after controlling for potential confounders.

## Conclusions

There exists a significant but small positive association between suicidal behaviors and comorbid ADHD in MDD adolescents. The suicide rate has increased by 52% from 2012 to 2014 in depressed adolescents with ADHD in the US. Due to higher impulsivity in MDD adolescents with ADHD, suicide risk is much higher. This calls for early diagnosis and management of ADHD and early-onset depression in adolescents to prevent suicide risk and mortality, and improve quality of life.

## References

[1] Birmaher B, Ryan ND, Williamson DE (1996). Childhood and adolescent depression: a review of the past 10 years. Part I. J Am Acad Child Adolesc Psychiatry.

[2] (2020). Major depression. https://www.nimh.nih.gov/health/statistics/major-depression.shtml.

[3] Thapar A, Collishaw S, Pine DS, Thapar AK (2012). Depression in adolescence. Lancet.

[4] (2020). Suicide. https://www.nimh.nih.gov/health/statistics/suicide.shtml.

[5] Balazs J, Kereszteny A (2017). Attention-deficit/hyperactivity disorder and suicide: a systematic review. World J Psychiatry.

[6] Lan WH, Bai YM, Hsu JW (2015). Comorbidity of ADHD and suicide attempts among adolescents and young adults with bipolar disorder: a nationwide longitudinal study. J Affect Disord.

[7] Dvorak RD, Lamis DA, Malone PS (2013). Alcohol use, depressive symptoms, and impulsivity as risk factors for suicide proneness among college students. J Affect Disord.

[8] Van Eck K, Ballard E, Hart S, Newcomer A, Musci R, Flory K (2015). ADHD and suicidal ideation: the roles of emotion regulation and depressive symptoms among college students. J Atten Disord.

[9] Danielson ML, Bitsko RH, Ghandour RM, Holbrook JR, Kogan MD, Blumberg SJ (2018). Prevalence of parent-reported ADHD diagnosis and associated treatment among U.S. children and adolescents, 2016. J Clin Child Adolesc Psychol.

[10] (2020). Overview of the national (nationwide) inpatient sample (NIS). https://www.hcup-us.ahrq.gov/nisoverview.jsp.

[11] (2020). NIS description of data elements. https://www.hcup-us.ahrq.gov/db/nation/nis/nisdde.jsp.

[12] Mowlem F, Agnew-Blais J, Taylor E, Asherson P (2019). Do different factors influence whether girls versus boys meet ADHD diagnostic criteria? Sex differences among children with high ADHD symptoms. Psychiatry Res.

[13] Morgan PL, Staff J, Hillemeier MM, Farkas G, Maczuga S (2013). Racial and ethnic disparities in ADHD diagnosis from kindergarten to eighth grade. Pediatrics.

[14] Winstanley CA, Eagle DM, Robbins TW (2006). Behavioral models of impulsivity in relation to ADHD: translation between clinical and preclinical studies. Clin Psychol Rev.

[15] Schatz DB, Rostain AL (2006). ADHD with comorbid anxiety: a review of the current literature. J Atten Disord.

[16] Wang PW, Yen CF (2017). Adolescent substance use behavior and suicidal behavior for boys and girls: a cross-sectional study by latent analysis approach. BMC Psychiatry.

[17] Patel RS, Patel P, Shah K, Kaur M, Mansuri Z, Makani R (2018). Is cannabis use associated with the worst inpatient outcomes in attention deficit hyperactivity disorder adolescents?. Cureus.

[18] Daviss WB (2008). A review of co-morbid depression in pediatric ADHD: etiologies, phenomenology, and treatment. J Child Adolesc Psychopharmacol.

[19] Huemer J, Riegler A, Volkl-Kernstock S, Wascher A, Lesch OM, Walter H, Skala K (2016). The influence of reported ADHD and substance abuse on suicidal ideation in a non-clinical sample of young men. Neuropsychiatr.

